# Polymorphisms in Pepsinogen C and miRNA Genes Associate with High Serum Pepsinogen II in Gastric Cancer Patients

**DOI:** 10.3390/microorganisms9010126

**Published:** 2021-01-07

**Authors:** Valli De Re, Mariangela De Zorzi, Laura Caggiari, Ombretta Repetto, Giulia Brisotto, Raffaela Magris, Stefania Zanussi, Agostino Steffan, Renato Cannizzaro

**Affiliations:** 1 Immunopathology and Cancer Biomarkers, Centro di Riferimento Oncologico di Aviano (CRO), IRCCS, 33081 Aviano, PN, Italy; mdezorzi@cro.it (M.D.Z.); lcaggiari@cro.it (L.C.); orepetto@cro.it (O.R.); gbrisotto@cro.it (G.B.); szanussi@cro.it (S.Z.); asteffan@cro.it (A.S.); 2 Oncological Gastroenterology, Centro di Riferimento Oncologico di Aviano (CRO), IRCCS, 33081 Aviano, PN, Italy; raffaella.magris@cro.it (R.M.); rcannizzaro@cro.it (R.C.)

**Keywords:** serum pepsinogen II, *PGC*, *Helicobacter pylori*, gastric cancer, polymorphisms, RNAs

## Abstract

Background: Pepsinogen (PG) II (PGII) is a serological marker used to estimate the risk of gastric cancer but how PGII expression is regulated is largely unknown. It has been suggested that PGII expression, from the *PGC* (Progastricsin) gene, is regulated by microRNAs (miRNA), but how PGII levels vary with *Helicobacter pylori (H. pylori)* infection and miRNAs genotype remains unclear. Methods: Serum levels of PGI and PGII were determined in 80 patients with gastric cancer and persons at risk for gastric cancer (74 first-degree relatives of patients, 62 patients with autoimmune chronic atrophic gastritis, and 2 patients with dysplasia), with and without *H. pylori* infection. As control from the general population, 52 blood donors were added to the analyses. Associations between PGII levels and genetic variants in *PGC* and miRNA genes in these groups were explored based on *H. pylori* seropositivity and the risk for gastric cancer. The two-dimensional difference in gel electrophoresis (2D-DIGE) and the NanoString analysis of messenger RNA (mRNAs) from gastric cancer tissue were used to determine the pathways associated with increased PGII levels. Results: PGII levels were significantly higher in patients with gastric cancer, and in those with *H. pylori* infection, than in other patients or controls. A PGI/PGII ratio ≤ 3 was found better than PGI < 25 ng/mL to identify patients with gastric cancer (15.0% vs. 8.8%). For two genetic variants, namely rs8111742 in miR-Let-7e and rs121224 in miR-365b, there were significant differences in PGII levels between genotype groups among patients with gastric cancer (*p* = 0.02 and *p* = 0.01, respectively), but not among other study subjects. Moreover, a strict relation between rs9471643 C-allele with *H. pylori* infection and gastric cancer was underlined. Fold change in gene expression of mRNA isolated from gastric cancer tissue correlated well with polymorphism, *H. pylori* infection, increased PGII level, and pathway for bacteria cell entry into the host. Conclusions: Serum PGII levels depend in part on an interaction between *H. pylori* and host miRNA genotypes, which may interfere with the cut-off of PGI/PGII ratio used to identify persons at risk of gastric cancer. Results reported new findings regarding the relation among *H. pylori*, PGII-related host polymorphism, and genes involved in this interaction in the gastric cancer setting.

## 1. Introduction

Gastric cancer (GC) is the third leading cause of tumor-related death worldwide [1]. Although its incidence is declining with the decline in the prevalence of its main risk factor, *Helicobacter pylori* (*H. pylori*) infection, GC remains a worrisome disease due to its poor prognosis and the increasing aging of the population [2,3]. *H. pylori* causes atrophic gastritis (AG) and intestinal metaplasia (IM), which are preneoplastic conditions to GC according to Correa’s cascade [4]. Another cause of AG is the development of autoantibodies against the H+ proton pump and parietal cells of the stomach. These autoantibodies are typical of autoimmune chronic atrophic gastritis (ACAG), another preneoplastic condition for GC development [5]. Finally, GC may be caused by genetic abnormalities. In particular, germline mutations in the *CDH1* gene are implicated in hereditary diffuse gastric cancer [6]. Surveillance of people at risk (i.e., individuals with chronic gastric atrophy or family history) to detect GC in its initial stages is fundamental to improve their survival. The use of serology combined with endoscopy and histological examination is a useful strategy for identifying persons at risk of developing GC [7,8]. Serological tests can measure pepsinogen I (PGI), pepsinogen II (PGII), gastrin-17, and IgG against *H. pylori* (e.g., [9]), independently or in combination, as in GastroPanel (Biohit, Helsinki, Finland). According to the British Society of Gastroenterology, patients who are found to have extensive AG or IM at screening endoscopy should undergo endoscopic surveillance every three years [10]. Endoscopic resection of gastric dysplasia and early GC can be curative and improve patient survival. For example, in Japan, resection of early GC provided a 90% 5-year survival rate compared to less than 30% for resection of stage III-IV GC [11].

Several cut-offs for serum PGI and PGI/PGII have been proposed as a screening test for chronic AG and identification of patients at risk for GC. A cutoff of PGI < 70 ng/mL and a PGI/PGII ratio ≤3 are the most widely used [12,13]. A recent meta-analysis reports for sensitivity and specificity values of 0.59 for detecting both AG and GC, and a specificity of 0.89 and 0.73, for AG and GC, respectively [14]. Lower PGI values i.e., ≤25 ng/mL [15] and <50 ng/mL [16] have also been proposed for predicting severe atrophy and IM.

PGI levels decrease with the extent of atrophy [17] and therefore lower the PGI/PGII ratio. A low PGI/PGII ratio may also be due to high PGII levels. PGII is produced by all sections of the stomach, while PGI is produced only by the corpus and fundus [17]. Under physiological conditions, PGII is mainly expressed in stomach tissue and is secreted into the stomach cavity by mature, differentiated mucosa cells; about 1% of PGII enters the blood circulation in a stable form through capillaries in the gastric mucosa [18,19]. PGII levels increase in patients with gastric ulcer or Zollinger-Ellison syndrome [20,21]. During *H. pylori* infection and inflammation PGII production also increases [22], resulting in higher PGII concentration in the blood and a reduction in the PGI/PGII ratio.

However, the mechanism, by which *H. pylori* mediates the increase of PGII levels is not fully understood. It is known that PGII is encoded by the *PGC* gene, which is regulated by promoter polymorphisms and miRNAs let-7e, miR-365b, and miR-4795 [23]. The promoter of each of these miRNAs contains a single nucleotide polymorphism (SNP) whose genotype was found to associate with GC in a Chinese case-control study [24]. Pairwise combinations of these SNPs and SNPs in the *PGC* promoter also associated with GC [23]. These studies, however, are limited to the Asian population and did not measure PGII levels, thus, the effect of the SNPs and *H. pylori* infection on PGII expression remained unclear. Therefore, in this study, we measured PGII levels in Italian patients with GC, in subjects at risk for GC, and controls, and then investigated if PGII levels associated with *H. pylori* infection, the genotypes of genetic variants in *PGC* and the three miRNAs.

## 2. Materials and Methods

### 2.1. Biological Materials and Research Ethics

This study continues ongoing research into GC and builds on an already published case series [16]. Briefly, between January 2013 and March 2018, patients are seen in the Oncological Gastroenterology unit for endoscopic resection of gastric mucosa for histologic evaluation were invited to donate a sample of venous blood for research purposes. This study uses 50 blood samples already analyzed in our previous study [16] and 220 not-yet analyzed samples, from patients with ACAG, GC, or high-grade gastric dysplasia, as well as from first-degree relatives of GC patients (FDR-GC). Besides, blood from blood donors was used as control samples.

### 2.2. Clinical Data and Analytical Procedures

Information on each patient’s diagnosis was collected. ACAG had been diagnosed both histologically, following the Updated Sydney System [25] for atrophy, and serologically according to the presence of autoantibodies against parietal cells with a cut-off for positivity of ≥1: 80 (APCA IgG, Euro-Immun, Lübeck, Germany). GC and Gastric dysplasia had been diagnosed by histological examination of endoscopic biopsies [26]. Gastric tissue from FDR-GC had been assessed by endoscopy and histological examination, and blood samples from these persons were included in the study if these analyses excluded ACAG, dysplasia, and GC. *H. pylori* infection was considered positive when the anti-*H. pylori* IgG titer was ≥30 EIU/mL (HP-IgG ELISA Biohit Healthcare).

Serum was obtained by centrifugation of venous blood at 2600× *g* for 10 min and stored at −80 °C until use. PGI, PGII, and gastrin-17 were measured using an ELISA kit (Biohit, Helsinki, Finland) and all blood samples were collected and process in the morning in fasting patients. The interval for the dosage was obtained by testing 200 blood donors. According to the parametric method, the 95% distribution reference value range is PGI 30–166 ng/mL, PGII 3–15 ng/mL, and G17 2.5–7 pmol/L.

### 2.3. Genotyping Assays

**Figure 1:**
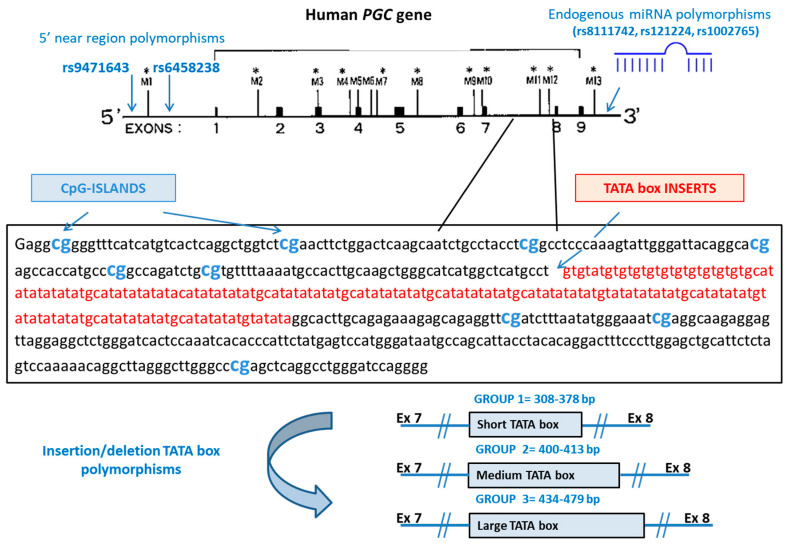
*PGC* (Progastricsin) gene, coding for the Pepsinogen II protein, structure and interactions with endogenous miRNAs tested in the study. The nine exons are indicated with black rectangles. * Potential methylation sites are indicated as M1 to M12. Different lengths of TATA box repeats (Group 1, short; Group 2, medium; Group 3, long) are present in the intronic region between exons 7 and 8, which includes two methylation sites (M11, M12) [29].

Genomic DNA was extracted from whole blood using the EZ1 DNA Blood kit (Qiagen, Hiden, Germany) and genotyped for six genetic variants (Table 1, Figure 1). These variants included two SNPs located upstream of the *PGC* transcriptional start site, in binding sites for transcription factors [27], three SNPs in three miRNAs predicted to regulate the expression of *PGC* gene by binding to its 3′UTR [24], and an insertion/deletion (ins/del) of varying length arising from the insertion of TATA boxes in the intron between exons 7 and 8 of the *PGC* gene [28].

SNP genotyping assays were performed using custom TaqMan genotyping assays (Applied Biosystems, Foster City, CA, USA), allele-specific oligonucleotide probes (Table 1), and dual-labeled primers, on a 7900HT Fast Real-Time PCR system (Applied Biosystems, Foster City, CA, USA). Genotypes were determined using allelic discrimination software (7300 System SDS software, Applied Biosystems, Foster City, CA, USA), and validated by amplicon sequencing.

Ins/del detection was done by amplifying the DNA fragment encompassing the TATA boxes using a fluorochrome (6 FAM)-labeled forward primer and an unlabeled reverse primer (Table 1) in a reaction of 25 μL including dNTPs (200 ng) and GoTaq DNA Polymerase (0.5 U, Promega, Madison, WI, USA) [30]. The amplification products were then sized on an ABI 3130 Genetic Analyzer along with molecular weight markers (LIZ 120 Size Standards) using GeneMapper Software v4.1 (all from Applied Biosystems, Foster City, CA, USA). Samples were assigned, according to the length of the TATA box-containing fragment, to one of three groups (Table 1, Figure 1).

### 2.4. Two-Dimensional Difference in Gel Electrophoresis, 2D-DIGE, Analysis

For the study, the corpus stomach biopsies of 8 patients with GC, 4 cases were *H. pylori*-positive, were analyzed by using the difference in gel electrophoresis (DIGE) of protein expression. Proteins were isolated, labeled with cyanine dyes and separated as reported in one of our previous papers [31]. From the protein map, the spot corresponding to the PGII protein was identified using the Decyder software and a liquid mass MS/MS spectrometry as previously reported [31]. The comparison of the PGII spots obtained from the GC proteome maps of every single patient and the same internal standard composed the standardized abundance ratio of PGII concentration in the tumor tissue. The internal standard corresponded to pooled equal amounts of 9 biopsies from the corpus of patients with autoimmune atrophic gastritis without GC, and all of the 8 GC sample studied.

### 2.5. NanoString Analysis

Total RNA from formalin-fixed, paraffin-embedded tissue sections of 13 patients with GC at diagnosis was extracted using an RNeasy DSP FFPE Kit (Qiagen, Hiden, Germany) according to the manufacturer’s protocol. DNase for optimized removal of eventual genomic DNA contamination was used. RNA quality was assessed using the High Sensitivity RNA ScreenTape kit (Agilent, Santa Clara, CA, USA) on the 2200 TapeStation system (Agilent, Santa Clara, CA, USA). RNA quantification was performed using the Qubit™ RNA HS Assay (Invitrogen, MA, USA) on the Qubit fluorometer 2.0. instrument (Invitrogen, MA, USA). Among these patients, 7 were H. pylori-positive. For each sample, 250 ng of RNA was hybridized with probes of a custom panel (Supplementary Table S1). The assay tested 38 genes representing GC related expression signature and 4 genes as housekeeping genes. Detection and scanning of RNA expression were acquired using the NanoString nCounter Analysis System (NanoString Technologies, Seattle, WA, USA) according to the manufacturer’s procedures. The background level of each tumor sample was obtained by subtracting the mean plus 2 standard deviations of the counts of the negative control probes comprised in the assay. Data were normalized using the geometric mean of the positive controls and the housekeeping genes included in the custom panel—B2M, GAPDH, HPRT1, and RPL19. Data analyses were performed using the nSolver 4.0 software (NanoString Technologies, Seattle, WA, USA). Log2 transformed expression data were fit to a linear model comprised of serum PGII levels and selected gene polymorphisms with H. pylori positivity as a covariable factor.

### 2.6. Statistical Analyses

The Shapiro–Wilk test was used to assess the normal distribution of the data. When values were normally distributed, ANOVA was used to compare quantitative variables among groups. The Kruskal–Wallis test was used when the assumption of normality was not confirmed. To test if medians were ordered, the Kruskal–Wallis test was following by the Jonckheere-Terpstra trend test and Conover post-hoc test to assess differences among groups or by Dunn’s test. Chi-square or Fisher’s exact test was used to analyze differences among groups for qualitative data and the chi-square for linear trends was used to assess differences in distributions of categorical variables among independent groups. Univariate and multivariate linear regression was done to identify clinical variables and genotypes associated with serum PGII levels. Receiver operating characteristic (ROC) curve analysis was used to extract a cut-off value PGII levels predictive of a positive IgG to *H. pylori* value, and to determine its sensitivity and specificity.

## 3. Results

### 3.1. Clinical Characteristics of Patients

The study used samples and data from blood donors (controls), first-degree relatives of patients with GC (FDR-GC), ACAG patients, patients with high-grade dysplasia, and GC patients, for a total of 270 study subjects (Table 2). There was a slight predominance of men among patients with GC (60%) while patients with ACAG were predominantly women (75.8%) (*p* < 0.0001, chi-square test). More than half of GC cases had *H. pylori* infection, about one-third of those at risk of GC (i.e., FDR-GC subjects and ACAG patients) were infected, and only 13.5% of controls had the infection.

Among GC patients, 53.7% had a diffuse and 36.2% had an intestinal histological type, and in 71.2% of cases, the disease was located distally (Table 3). An advanced stage at presentation was recorded in 61.2% of cases, and 55.0% had *H. pylori* infection.

### 3.2. Trends in Pepsinogen I and II Levels

**Figure 2:**
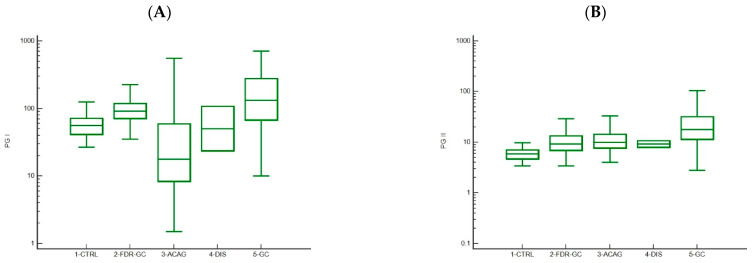
Serum levels of pepsinogen I and II, by study group. The boxes indicate the median and 25th and 75th percentiles, and the outside whiskers indicate the range. (**A**) Pepsinogen I. Kruskal-Wallis test, *p* < 0.0001, Jonckheere-Terpstra test for trend not significant. (**B**) Pepsinogen II. Kruskal-Wallis test, *p* < 0.0001, Jonckheere-Terpstra test for trend *p* < 0.0001, significantly different (*p* < 0.05) average rank (Conover analysis). CTRL, controls; FDR-GC, first-degree relatives of gastric cancer patients; ACAG, autoimmune chronic atrophic gastritis; DIS, dysplasia; GC, gastric cancer.

Compared with PGI levels in controls (median, 56.25 ng/mL [IQR, 41.5–71.4]), median PGI levels were lower in ACAG patients (17.8 ng/mL; IQR, 8.5–67.6) and in patients with high-grade dysplasia (65.9 ng/mL; IQR, 23.7–108.0) and higher in FDR-GC (91.4 ng/mL; IQR, 77.0–123.7) and GC patients (132.8 ng/mL; IQR, 68.1–225.5) (Figure 2A)**.** Regarding PGII (Figure 2B), the lowest median level was found in controls (5.9 ng/mL, IQR 5.2–11.0), and progressively higher median levels were recorded in patients at risk for GC (i.e., FDR-GC, 9.3 ng/mL [IQR, 11.0–28.7]; ACAG, 10.0 ng/mL [IQR, 7.0–15.1]; high-grade dysplasia, 9.4 ng/mL [IQR, 8.0–10.7]); the highest levels were found in GC patients (17.8 ng/mL [IQR, 11.0–28.7]) (Kruskal-Wallis test for trend, *p* < 0.001).

We next explored the usefulness of serum marker cut-offs to predict gastric disease or risk of disease (Table 4). Using a cut-off of PGI <25 ng/mL as a biomarker for GC risk, we found that all controls had normal levels; instead, one FDR-GC and one patient with high-grade dysplasia had levels below the cut-off, 59.7% of ACAG patients had abnormally low values. Regarding the PGI/PGII ratio and using a cut-off of ≤3 as pathological, we again found that all controls had normal values; one FDR-GC and both patients with dysplasia had abnormally low values, as did 66.1% of AGAC patients. For GC patients, only 7 (8.8%) had abnormally low levels of PGI by the 25 ng/mL cut-off, whereas 12 patients (15.0%) had an abnormally low PGI/PGII ratio. This difference can be attributed to the high PGII level.

**Figure 3:**
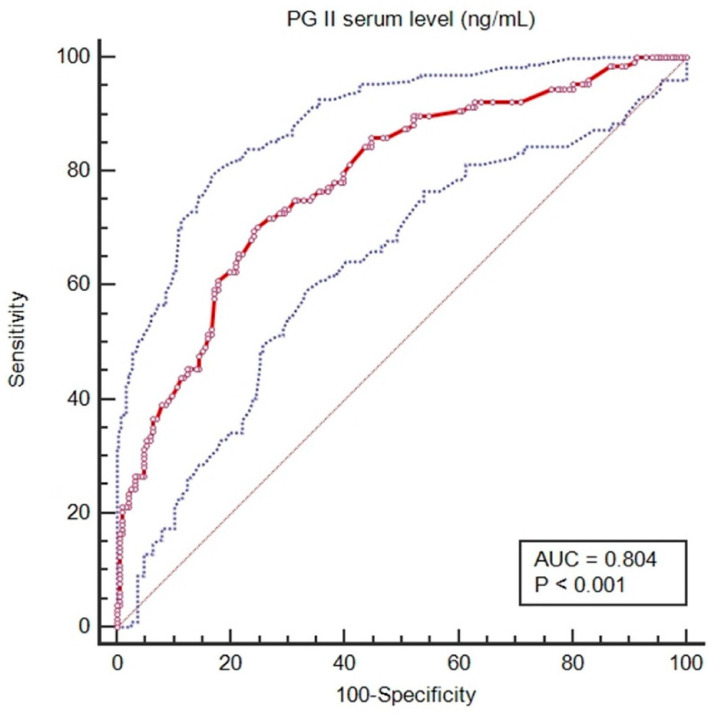
Receiver operating characteristic curve for predicting the presence of anti-*H. pylori* IgG from serum Pepsinogen (PG) II levels.

To investigate the regulation of PGII levels, multivariate logistic regression was done using PGII in GC patients as the dependent variable and GC localization, age at diagnosis, sex, clinical stage, histological type, and anti-*H. pylori* IgG seropositivity as the independent variables. Only anti-*H. pylori* IgG seropositivity was retained as an independent variable associated with serum PGII (*p* < 0.0001). By receiver operating characteristic (ROC) curve analysis, anti-*H. pylori* IgG seropositivity was confirmed to be an important variable for serum PGII levels in all study subjects considered together. The area under the ROC curve (AUC) was 0.804 (95% CI, 0.751 to 0.850; *p* < 0.001; Figure 3). Setting a cut-off of PGII >13 ng/mL to define *H. pylori* infection gave a 68.8% sensitivity and an 81.1% specificity.

**Figure 4:**
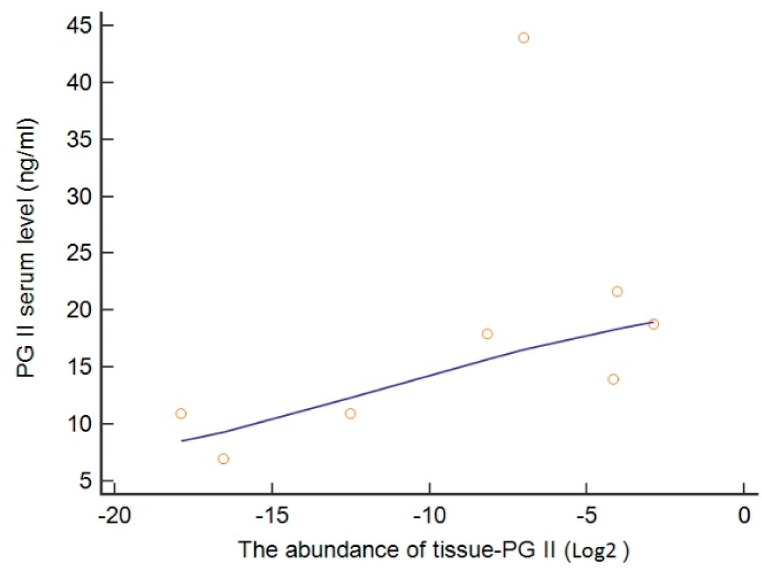
Positive relationship between serum PGII levels and abundance of PGII from the paired patient samples.

**Figure 5:**
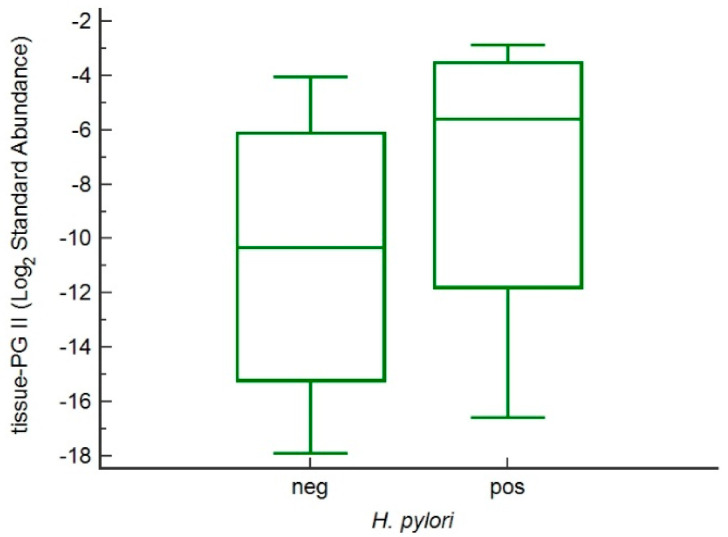
Box plot showing median increased of PGII standard abundance in *H. pylori*-related GC tissue specimens.

The serum PGII levels correlated well with PGII tissue protein concentration obtained by 2D-DIGE analysis from paired patient’s samples (*p* = 0.035, Figure 4). The median PGII concentration from tumor biopsies were found higher in *H. pylori*-positive than in *H. pylori*-negative samples like from serum PGII samples (Figure 5).

Nanostring analysis of normalized data mRNAs obtained from GC tissue samples identified twenty-one genes significantly associated with increased serum PGII levels (Table 5).

**Figure 6:**
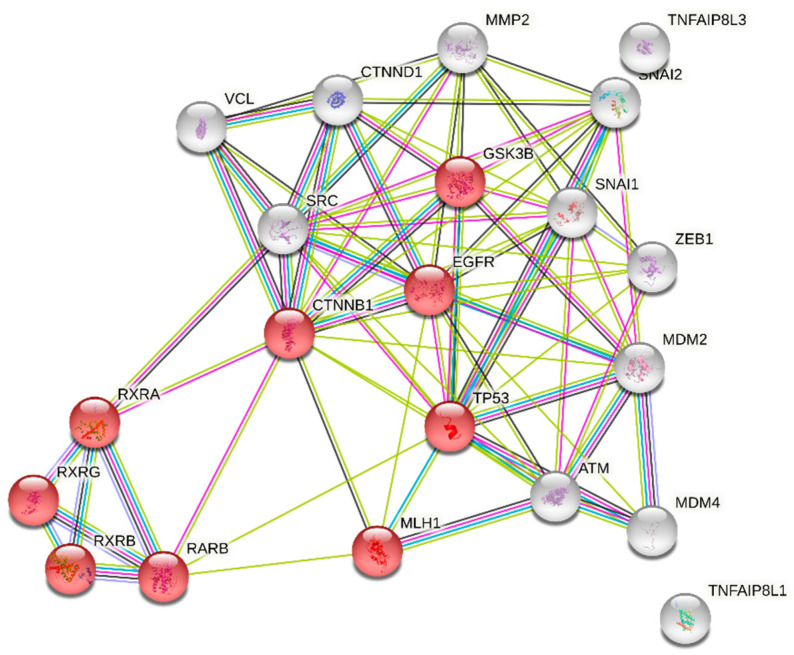
PG II-related protein-protein interaction network generated using STRING. The network is made up of the 21 significant genes (Benjamin corrected *p* < 0.05) reported in Table 5. Colored nodes indicated the first choice of interactors.

Using selected genes reported in Table 5, STRING pathway analysis consistently identified the most meaningful KEGG pathway, which was related to the gastric cancer signaling network (hsa05226, count 9/147 false discovery rate (FDR) 2.99 × 10^−12^ Figure 6).

### 3.3. Genetic Regulation of PGII Levels

First, we investigated the effect of the genotype of six variants on serum PGII levels in all study subjects (Table 6). Only a significant effect (*p* = 0.03, Kruskal-Wallis test) was found for rs9471643, with higher PGII levels in the C/C group. However, after stratification by *H. pylori* infection on the study group, the rs9471643 genotype did not associate with PGII concentration (Supplementary Table S2) and PGII levels progressively increased with the progression of gastric disease in all the rs9471643 genotypes (Supplementary Table S3). Thus, the higher PGII concentration associated with the rs9471643 C/C genotype is due to the higher proportions of *H. pylori*-positive cases and GC cases with the C/C genotype.

After *H. pylori* stratification the number of cases and serum PGII levels in GC groups were compared to controls (Table 7). CG genotype resulted associated with a significant increase in the number of cases (RR 1.40) and PGII expression (2.59 fold) in *H. pylori*-positive gastric cancer, while CG genotype was not found related to *H. pylori*-positive controls.

NanoString analysis of mRNAs from GC tissue samples identified eleven genes associated with the rs9471643 C-allele (*p* < 0.05), although after multiple gene Benjamin correction none of them retained significant value (Table 8).

**Figure 7:**
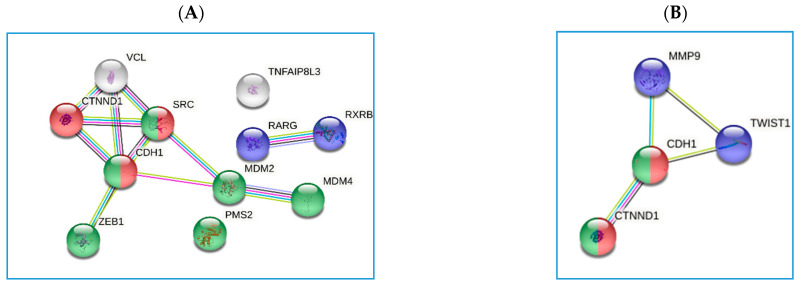
Polymorphism-related protein-protein interaction networks generated using STRING. Colored nodes indicated the first choice of interactors. (**A**) The network is made up of the 11 genes (*p* < 0.05) reported in Table 8 for the rs9471643 SNPs. (**B**) The network is made up of the 4 genes reported in Table 7 for the pre-miRNA rs8111742 and rs121224 SNPs.

To explore the interaction between the differentially expressed genes reported in Table 8, we constructed a protein-protein interaction network using the STRING database. The returned protein interaction analysis is depicted in Figure 7. With regards to the rs9471643 C-allele, the main interactions found in the network were related with the entry of bacterium into the host cell (GO:0035635, FDR 2.46 × 10^−5^), molecular events in primary and metastatic colorectal carcinoma (PMID:22997602, FDR 6.62 × 10^−7^), and Retinoic acid receptor, and Retinoid X receptor (CL:12071, FDR 3.12 × 10^−5^ pathways (Figure 7A), while, concerning the rs8111742 and rs121224 SNPs, we found an association with entry of bacterium into the host cell (GO:0035635, FDR 0.00091), and positive regulation of DNA binding (GO:0043388, FDR 0.0149) pathways (Figure 7B).

We then analyzed the effect of genotype on PGII levels in the 80 GC patients and, separately, in the 190 other study subjects (herein named “non-GC”). This analysis identified significant differences in PGII levels in two genotype groups in GC (Table 9). For rs8111742 (in the miR-Let-7e gene), median PGII levels were lower in GC cases with the G/G genotype and with the G allele. For rs121224 (in the miR-365b gene), median PGII levels were higher in GC cases with the C/C genotype and the C/G genotype. No significant differences were found for these SNPs or the other tested SNPs among the non-GC study subjects.

In GC patients stratified based on *H. pylori* infection (Table 10), only the rs8111742 *PGC* genotyping showed a significant association with *H. pylori* seropositivity and a higher PGII increased level in patients having the rs8111742 A allele.

Since rs8111742 and rs121224 are in miRNAs that bind the same 3′UTR of *PGC* gene, data suggest that this region may have a role in the serum PGII increase level mediated by *H.pylori* infection in GC.

The relationship between both rs8111742 A- allele (pri-miR-Let-7e gene) and rs121224 G-allele (pri-miR-365b) in individuals based on GC disease and *H. pylori* infection (Figure 7B) identified the same pathways of an entry of bacterium into the host cell (GO:0035635, FDR 0.00091), and positive regulation of DNA binding (GO:0043388, FDR 0.0149).

This analysis confirmed the positive trend between an increase in PGII level and increased risk for GC, in particular in subjects having an *anti-H. pylori* seropositivity and specific PGII-related polymorphisms.

## 4. Discussion

Identifying people at high risk for GC is a priority to increase the patient’s survival. A reduced PGI/PGII ratio is known to be associated with an increased risk of GC. However, confounding factors of PGII levels may distort the true association of PGII level and risk for GC.

This study found that patients with GC have higher PGII levels than persons at risk of GC. PGII levels were also significantly higher in GC patients with *H. pylori* infection than in those without. Finally, there were significant differences in PGII levels in subgroups of GC patients, and in particular in those with *H. pylori* infection, according to rs9471643 C -allele and rs81117242 SNPs in miR-Let-7e gene thought to regulate the *PGC* gene.

In our series, a PGI/PGII ratio ≤3 identified more patients with GC than did a cut-off of <25 ng/mL PGI, which better defines atrophy in the corpus. This observation is in line with the highest serum PGII levels being recorded in GC patients compared to individuals at risk for GC (i.e., FDR-GC and ACAG patients) and controls. A high PGII level reduces the PGI/PGII ratio, resulting in more GC patients falling under the PGI/PGII ratio cut-off ≤3. Thus, combining PGII and PGI was the best serological test for GC risk.

High levels of PGII are related to the presence of *H. pylori* infection in GC cases. A previous in vitro study demonstrated that *H. pylori* lipopolysaccharide-induced the release of PGII from guinea pig gastric mucosa [32]. Other studies have shown that serum PGII concentrations correlate with the extent of the *H. pylori*-induced gastric mucosal damage and risk of GC [20,32,33,34,35,36]. Correa’s cascade (i.e., *H. pylori*-induced gastritis, atrophy, metaplasia, dysplasia, and then GC) is the main model of GC development. This model is principally valid for intestinal-type GC, but several studies have found that diffuse-type GC is also associated with *H. pylori* infection even if it does not follow Correa’s cascade [37,38,39]. Overall, our data confirm that *H. pylori* infection is associated with GC of different histological types.

A direct role of PGII in GC development has been hypothesized [18,19]. PGII protein in the stomach participates to maintain and protect normal cellular morphology by increasing the digestive power and thus acting to move away exogenous risky compounds and accelerate the stomach emptying [18]. Thus, higher PG II concentration might result in a defensive factor against injuries and harmful products release during infection. Nonetheless, the ability of PGII to degrade the extracellular matrix and act as an antimicrobial agent has suggested that it may also stimulate tumor growth [19,40]. High PGII expression in tumors in anatomical sites other than the stomach (e.g., prostate, breast, ovary, and endometrium) supports a possible role of PGII in cancer [19,40].

Several animal models and epidemiological studies, including a recent study of Yuan et al. [36], demonstrated clinical relevance of *H. pylori* infection in gastric diseases and stepwise progression towards GC, and further, they found that infection increased the PG II concentration in the peripheral blood. Another study in a large series of cases reported an increase of GC development in patients with *H. pylori* infection and high-titer of PG II level, and conversely, a low incidence rate in subjects stratified with low serum levels of both PG II and *H. pylori* antibodies [41]. Overall data support an association between an increase in PG II level and GC development; however, its putative role in GC pathogenesis remains unclear and requires further studies.

This study found that three SNPs were associated with serum PGII levels.

The G>C transversion at rs9471643 in the *PGC* gene has already been reported to play a role in activating the gene’s promoter, leading to an increase in PG II expression and a change in the nuclear transcription factor binding ability (nuclear mobility shift) [27]. The same authors found a lower number of rs9471643 CG than GG genotype in cases vs. controls, suggesting a protective role of rs9471643 CG genotype on GC development.

Our data indicated a median highest serum PG II levels in overall subjects having the rs9471643 CC genotype compared to the GG genotype, however, we found that the increased PG II level was more dependent on *H. pylori*-positivity and patient’s disease (Supplementary Tables S1 and S2). We demonstrated a good relation of PGII levels between serum and GC tissue specimens, and accordantly PGII levels both in serum and tissue specimens were found to increase in GC *H. pylori*-positive cases. Moreover, the relative risk of *H. pylori*-positive GC was higher in patients having the CC and CG genotypes than in patients having the GG genotype, while we did not find any *H. pylori*-positive CTRL with the CG genotype (Supplementary Table S3). Besides, the study demonstrated a strict relation between an increase in PGII levels and GC-related mRNA expression signature, with most of these mRNA genes showing a different tissue expression between rs9471643 allele GC subtypes. Of note, these genes were enriched for entry of bacterium into the host and of the KEGG gastric cancer pathway, and thus supported the association of *H. pylori* infection and rs9471643 C allele with the increase of PG II level like a risk marker for GC.

Another SNP associated with PGII levels was rs8111742 in miR-Let-7e, an endogenous miRNA predicted to bind the 3′-UTR of *PGC* [23,42]. This miRNA is a direct regulator of numerous genes involved in cell proliferation and differentiation, and it shows a tumor suppressor function for several oncogenes (e.g., *RAS*, *MYC*), which may explain why a reduction in its expression in a variety of cancers has been associated with poor prognosis and increased epithelial-mesenchymal transition [43,44,45,46]. Due to its important role in both physiology and disease, miR-Let-7e is now considered a target for therapeutic drug development [47]. Two studies have reported a reduction of miR-Let-7e expression in the tissue of GC patients [48,49], and lower levels in GC patients with *H. pylori* infection than controls [50]. Thus, the miR-Let-7e reduction is a common event in GC. We found an association between lower serum PGII and the G/G genotype of rs8111742 in all GC patients and the subgroup of GC patients without *H. pylori* infection. These findings indirectly support a role in miR-Let-7e in GC pathogenesis *H. pylori*-mediated.

The third SNP associated with serum PGII levels was rs121224 in miR-365b. This miRNA suppresses tumor cell growth and invasion of cancers such as breast cancer, hepatocarcinoma, small cell lung cancer, and GC [51,52,53,54]. In some tumors, serum miR-365 is a diagnostic or prognostic marker [54,55,56,57]. In GC, activation of Akt signaling pathway was found to decrease the expression of miR-365, and consequently to promote cell growth by increasing the expression of cyclin D1 and CDC25A [52]. Akt signal activation is best understood as the major cascade stimulating the epithelial-mesenchymal transition-related to cell migration and invasion, and the most common molecular finding in various human cancers, including GC. Since GC is characterized by high cell proliferation via the Akt pathway [58], this pathway is a potential therapeutic target.

We found an association between serum PGII levels and the genotype of rs121224 in miR-365b, limited to GC patients, with high PGII levels in those with the C/C or C/G genotype and low levels in those with G/G. The G/G genotype was previously associated with a better prognosis in GC [24]. Thus, we suggest that the G/G genotype may have a protective role in GC. We also found that the association with genotype was lost in GC patients with *H. pylori* infection. Serum PGII level was higher in GC patients with *H. pylori* infection than in those without, in all the genotype subgroups. Therefore, the rs121224 polymorphism may regulate *PGC* expression, and factors directly related to *H. pylori* infection or produced by GC cells in subjects *H. pylori*-infected may interfere with miR-365—*PGC* interactions. Moreover, differentially expressed genes from the rs8111742 and the rs121224 specimen subtypes were both enriched for the entry of bacterium into host cell/cell adhesion molecule binding and positive regulation of DNA binding.

In this contest, in recent years in addition to genetic alterations, several cancer cells change in the epigenome have been reported, including methylation gene due to *H. pylori* infection and/or miRNAs that can interact with nuclear DNA to direct localization of genes concomitantly involved in several pathways to control the production of proteins that may be involved also in different cancer types. Thus, a perturbation in miRNAs expression due to genetic modification in pre-miRNA region linking to specific regulatory gene expression may produce the acquisition of new cellular characteristics as a different response to treatment or tumor hypoxia, or directly to PGII expression [59,60].

## 5. Conclusions

The PGI/PGII ratio was better than PGI alone to identify patients with GC, due to higher serum levels of PGII in these patients than in the other study groups. Higher serum PGII is not only dependent on *H. pylori* infection and atrophy but also polymorphisms in the PGII promoter region, miR-Let-7e, and miR-365b genes. Overall genes-related to polymorphisms in rs9471643, miR-Let-7e, and miR-365b were found associated with the pathways involved in bacteria entry into the host and with genes found associated with increased PGII levels-related to gastric cancer.

Further studies are required to elucidate the role of *H. pylori* in combination with PGII-related SNPs in GC carcinogenesis.

## Figures and Tables

**Table 1:** Genetic variants tested for possible roles in regulating the expression of the *PGC* (Progastricsin) gene encoding pepsinogen II.

Variant	Gene Region	Change	Probes and Primers
rs9471643	5′UTR of PGC	C > G	AGATTTGAACATAGCTGTGATCGTT[C/G]TGTAAAGGCAACTTCTGTTTTGCCC
rs6458238	5′UTR of PGC	A > G	GGTGGTGCCCAGGCTTTTTCTTATC[A/G]CCTTCTAGTCTCATTTCCTTTAAGC
rs8111742	5′UTR of miR-Let-7e	A > G	AGGTGTGCCCAAAGGGCCAAATTAC[A/G]AGACAAATGAGGTTCCCTCCCAGCC
rs121224	5′UTR of miR-365b	C > G	AAGAAGGTTGGAGGCTGGCTGTCTT[C/G]TGTTCATGTGTCACGCCAGGCCTGA
rs1002765	5′UTR of miR-4795	A > G	CCTCTGCTTCGATTGTTGCTCTTCA[A/G]TTGGGCGATATACTACTTGTAGGCA
Ins/del of TATA boxes	PGC intron between exons 7 and 8	308–378 bp 400–413 bp 434–479 bp	Forward 5′-6-FAM-GGCCAGATCTGCGTGTTTTA-3′ Reverse 5’-AGCCCTAAGCCTGTTTTTGG-3′

UTR, untranslated region, Ins/del, insertion/deletion.

**Table 2:** Baseline characteristics of study subjects, by study group.

Characteristic	Controls (n = 52)	FDR-GC (n = 74)	ACAG (n = 62)	Dysplasia (n = 2)	GC (n = 80)
Age, years, mean (±SD)	56 (12)	47 (14)	52 (11)	58.2 (12.9)	61 (12)
Male, n (%)	37 (71.2)	30 (40.5)	15 (24.2)	0 (0)	48 (60.0)
***H. pylori* infection**					
Positive cases, n (%)	7 (13.5)	22 (29.7)	20 (32.3)	1 (50.0)	44 (55.0)
IgG, IU/mL, mean (±SD) §	76 (28)	89 (26)	85 (33)	57.4 (--)	90 (28)

SD, standard deviation; ACAG, patients with autoimmune chronic atrophic gastritis; FDR-GC, first-degree relatives of patients with gastric cancer; GC, gastric cancer; SD, standard deviation § Calculated only for those cases with *H. pylori* infection.

**Table 3:** Clinical characteristics of the subgroup of 80 patients with gastric cancer.

Characteristic	n (%)	PGII (ng/mL) §	PGI/PGII Ratio §
**Histotype**			
Intestinal	29 (36.2)	14.2 (8.4–23.3)	7.3 (5.1–10.7)
Diffuse	43 (53.7)	16.0 (9.9–28.2)	7.1 (3.9–10.0)
Mixed	8 (10.0)	18.8 (16.2–23.9)	7.3 (5.9–9.2)
**Location**			
Proximal	23 (28.7)	19.5 (9.5–33.4)	8.5 (5.4–11.6)
Distal	57 (71.2)	16.0 (11.0–22.3)	7.1 (4.3–10.0)
**Clinical stage**			
I-II	31 (38.8)	17.0 (10.0–22.8)	7.3 (5.9–11.3)
III-IV	49 (61.2)	16.0 (12.1–32.6)	7.1 (4.2–9.8)
***H. pylori*** **infection**			
Yes	44 (55.0)	19.9 (15.0–39.4)	7.0 (4.8–9.0)
No	36 (45.0)	12.6 (7.2–19.1)	8.5 (5.4–12.9)
*p* *		<0.001	0.044

§ median (IQR); * HP-IgG ELISA Biohit Healthcare for *H. pylori* infection, yes vs. no.

**Table 4:** Serum markers, by study group.

Serum Marker	Controls (n = 52)	FDR-GC (n = 74)	ACAG (n = 62)	Dysplasia (n = 2)	GC (n = 80)
PGI, ng/mL ^a^	56.25 (41.5–71.4)	91.4 (77.0–123.7)	17.8 (8.5–67.6)	65.9 (23.7–108.0)	132.8 (68.1–225.5)
PGI <70 ng/mL, n (%)	38 (73.1)	14 (18.9)	48 (77.4)	2 (100)	23 (28.7)
PGI <25 ng/mL, n (%)	0 (0)	1 (1.4)	37 (59.7)	1 (50.0)	7 (8.8)
PGII, ng/mL ^a^	5.9 (5.2–11.0)	9.3 (11.0–28.7)	10.0 (7.0–15.1)	9.4 (8.0–10.7)	17.8 (11.0–28.7)
PGI/PGII ratio ^a^	9.5 (8.2–13.1)	10.5 (7.86–13.04)	2.1 (1.01–5.7)	2.8 (2.7–3.0)	7.1 (4.9–10.1)
PGI/PGII ratio ≤3, n (%)	0 (0)	1 (1.4)	41 (66.1)	2 (100)	12 (15.0)
Gastrin-17, pmol/L ^a^	2.0 (0.7–8.1)	4.3 (1.7–13.4)	105.9 (27.3–248.5)	88.9 (1.94–175.9)	14.6 (3.8–26.9)

ACAG, patients with autoimmune chronic atrophic gastritis; FDR-GC, first-degree relatives of patients with gastric cancer; GC, gastric cancer; PGI, pepsinogen I; PGII, pepsinogen II. ^a^ Median (IQR).

**Table 5:** Fold change mRNA gene expression associated with increased serum PGII levels.

Gene Name	Log2 Fold Change	Std Error (log2)	*p*-Value	*p*-Corrected *
MDM2	0.118	0.0204	0.000000567	0.000736
SNAI2	0.089	0.0163	0.0000133	0.000863
EGFR	0.0858	0.0165	0.0000024	0.00107
MMP2	0.106	0.0228	0.000010	0.00326
RARb	0.0704	0.016	0.000186	0.00482
TIPE1 or TNFAIP8L1	0.0903	0.021	0.000241	0.00521
CTNNB1	0.062	0.0147	0.000305	0.00567
RXRg	0.126	0.0308	0.000404	0.00656
TIPE3 or TNFAIP8L3	0.111	0.0286	0.000696	0.00972
ATM	0.055	0.0143	0.000776	0.00972
VCL	0.0867	0.0227	0.000823	0.00972
MDM4	0.0626	0.0167	0.000986	0.0104
RXRb	0.0622	0.0167	0.00104	0.0104
CTNND1	0.0606	0.0166	0.00129	0.012
SNAI1	0.079	0.0226	0.00191	0.0165
ZEB1	0.0921	0.0274	0.00262	0.0213
SRC	0.0566	0.0175	0.00346	0.0265
GSK3B	0.0538	0.0167	0.00373	0.0269
MLH1	0.0574	0.0187	0.00525	0.0359
RXRa	0.0557	0.0192	0.00787	0.0499
TP53	0.05	0.0173	0.00806	0.0499
RARg	0.0548	0.0201	0.0119	0.0704
MSH6	0.0459	0.0177	0.0161	0.0911
RARa	0.0425	0.0167	0.0174	0.0943
PMS2	0.0409	0.0167	0.0217	0.113
CDH1	0.0341	0.0146	0.0281	0.14
HER2	−0.0578	0.0264	0.0387	0.186

* Benjamini-Yekutieli adjustment.

**Table 6:** Association of genotype with serum PGII levels in all 270 study subjects.

Variant	Genotype	Cases, n	PGII, ng/mL §	*p* *
rs9471643	C/C	18	16.6 (9.1–22.4)	0.03
	C/G	90	9.0 (6.4–15.6)	
	G/G	162	10.1 (6.3–17.1)	
rs6458238	A/A	3	4.5 (4.0–7.2)	0.61
	A/G	36	9.4 (7.6–15.1)	
	G/G	231	10.1 (7.7–18.7)	
rs8111742	A/A	19	10.1 (8.3–18.9)	0.06
	A/G	113	12.0 (6.7–18.5)	
	G/G	138	9.0 (6.3–14.0)	
rs121224	C/C	91	10.5 (6.9–16.5)	0.08
	C/G	124	10.3 (6.5–18.7)	
	G/G	55	11.65 (8.6–36.0)	
rs1002765	A/G	58	11.3 (6.4–17.6)	0.31
	G/G	212	9.6 (6.7–16.2)	
Indel (TATA boxes)	Group 1 (308–378 bp)	78	11.3 (7.4–19.0)	0.07
	Group 2 (400–413 bp)	25	9.5 (6.5–13.1)	
	Group 3 (434–479 bp)	169	12.1 (7.9–18.8)	

§ Median (IQR); * Kruskal-Wallis test.

**Table 7:** Rs9471643 CC and CG genotypes are associated with the highest relative risk for *H. pylori*-positive gastric cancer, and CG genotype also with the highest increase in PGII serum level. By converse, the CG genotype is not found in H. pylori-positive controls.

rs9471643	GC *H. pylori*-*positive*		GC *H. pylori*-*negative*				
	n	PGII §	n	PGII §	RR (95%CI) *p*	Fold Change	*p* &
C/C	7	22.4 (17.5–41.5)	1	22.1 (22.1–22.1)	1.57 (1.13–2.20) <0.01	1.01	0.82
C/G	17	18.8 (15.7–28.4)	6	7.1 (5.0–20.0)	1.40 (0.99–1.98) 0.05	2.59	0.05
G/G	23	27.1 (16.2–39.8)	26	12.8 (9.5–19.4)	0.6 (0.43–0.86) <0.01	2.11	<0.0001
*p* *		0.46		0.31			
**rs9471643**	**CTRL *H. pylori*** **-*positive***		**CTRL *H. pylori*** **-*negative***				
	**n**	**PGII §**	**n**	**PGII §**	**RR (95%CI) *p***	**Fold Change**	***p*** &
C/C	2	6.6 (6.6–6.6)	4	6.5 (6.1–6.9)	2.72 (0.78–9.50) 0.12	1.02	1.00
C/G	0	---	36	5.8 (4.7–6.8)	0.06 (0.01–1.05) 0.05	---	---
G/G	12	9.0 (8.3–14.0)	50	5.6 (4.7–6.1)	4.06 (0.96–17.24 0.06	1.60	0.001
*p* *		0.14		0.31			

§ Median (IQR); & Kruskal-Wallis test, * Jonckheere-Terpstra trend test. RR, the relative risk.

**Table 8:** Fold change mRNA gene expression associated with PGII and miRNA gene related polymorphisms.

Polymorphisms	Gene Name	Log2 Fold Change	Std Error (log2)	*p*-Value
rs9471643 C*vs*G allele	MDM2	−2.40	0.905	0.0143
	SRC	−1.76	0.693	0.0181
	RXRb	−1.57	0.632	0.0207
	CDH1	−1.29	0.523	0.0217
	CTNND1	−1.59	0.689	0.0304
	VCL	−2.19	0.955	0.0312
	PMS2	−1.37	0.616	0.0366
	TIPE3 or TNFAIP8L3	−2.68	1.21	0.0367
	ZEB1	−2.50	1.13	0.0377
	MDM4	−1.51	0.7	0.0418
	RARg	−1.71	0.809	0.0457
rs8111742 G*vs*A allele	TWIST1	1.10	0.771	0.167
	CTNND1	−0.69	0.551	0.224
rs121224 G*vs*C allele	MMP9	−1.33	0.506	0.015
	CDH1	−0.72	0.316	0.0319

**Table 9:** Effect of genotype and allele on PGII levels in 80 patients with gastric cancer (GC) and the other 190 study subjects.

Variant	GC		Non-GC		
	Cases, n	PGII, §	Cases, n	PGII, §	*p* *
rs8111742					
A/A	5	19.4 (11.6–67.6)	14	9.8 (7.6–16.8)	0.06
A/G	38	20.1 (15.0–39.4)	76	10.9 (7.1–15.0)	<0.0001
G/G	37	15.4 (8.6–22.2)	100	9.0 (7.1–12.6)	<0.001
*p* *		0.02		0.48	
A	48	19.8 (15.0–40.5)	104	9.0 (5.7–14.3)	<0.0001
G	112	17.0 (10.0–24.9)	276	8.0 (6.5–11.5)	<0.0001
*p* *		0.02		0.47	
rs121224					
C/C	31	16.0 (11.0–22.2)	61	10.6 (7.2–15.0)	<0.0001
C/G	36	20.3 (15.5–39.8)	88	9.8 (6.9–14.3)	<0.0001
G/G	15	11.0 (6.4–21.8)	41	9.2 (7.5–11.7)	0.24
*p* *		0.01		0.47	
C	98	17.3 (12.6–33.7)	210	8.0 (5.6–12.8)	<0.0001
G	62	18.3 (9.5–29.0)	170	8.1 (6.1–11.3)	<0.0001
*p* *		0.55		0.67	

* Kruskal-Wallis test, post-hoc analysis with Dunn’s test; § ng/mL, median (IQR).

**Table 10:** Effect of genotype and allele on PGII levels in 80 patients with gastric cancer (GC) based on anti-*H. pylori* seropositivity.

Variant	*H. pylori*-*positive*		*H. pylori-negative*		
	Cases, n	PGII, §	Cases, n	PGII, §	*p* *
**rs8111742**					
A/A	1	104 (104–104)	4	16.0 (10.8–37.4)	0.16
A/G	23	27.1 (17.7–42.2)	15	17.0 (12.7–20.2)	0.006
G/G	23	18.3 (14.4–34.0)	14	6.0 (3.9–11.0)	0.0002
*p* *		0.03		0.03	
A	25	33.7 (18.2–43.0)	23	17.0 (12.5–20.5)	<0.0001
G	69	20.3 (15.9–37.1)	43	10.8 (4.6–17.1)	<0.0001
*p* *		0.04		0.006	
**rs121224**					
C/C	18	19.5 (15.4–40.0)	13	12.5 (4.7–17.6)	0.005
C/G	25	20.6 (18.1–41.8)	11	17.1 (12.7–20.15)	0.006
G/G	4	20.5 (12.9–30.1)	9	7.2 (4.6–14.4)	0.002
*p* *		0.58		0.053	
C	61	20.6 (16.8–37.6)	37	13.0 (9.4–19.4)	<0.0001
G	33	20.6 (16.0–40.3)	29	12.0 (7.0–21.7)	<0.0001
*p* *		0.99		0.76	

* Kruskal-Wallis test, post-hoc analysis with Dunn’s test; § ng/mL, median (IQR).

## Data Availability

Data is contained within the article.

## Supplementary Materials

The following are available online at https://www.mdpi.com/2076-2607/9/1/126/s1, Table S1: List of custom probes used for the nanostring mRNA gene expression analysis; Table S2: Considering all subjects, higher PGII serum level is associated with H. pylori infection, independently of rs9471643 polymorphism genotype; Table S3: PGII serum level shows an increasing trend in preneoplastic diseases and GCs.
