# Supplementary material for: Polymorphisms in Pepsinogen C and miRNA Genes Associate with High Serum Pepsinogen II in Gastric Cancer Patients
Source: Microorganisms. 2021 Jan 7;9(1):126. doi: 10.3390/microorganisms9010126 (PMC7827830; doi:10.3390/microorganisms9010126)
Supplement: Supplementary file 1 [file microorganisms-09-00126-s001.pdf]

Supplementary Table S1. List of custom probes used for the NanoString mRNA gene expression analysis.

Gene	Accession Number	Class Name
ATM	NM_138292.3	Endogenous
CDH1	NM_004360.2	Endogenous
CTNNB1	NM_001098210.1	Endogenous
CTNND1	NM_001331.2	Endogenous
EBER1	KP195701.1	Endogenous
EGF	NM_001963.4	Endogenous
EGFR	NM_201282.1	Endogenous
GSK3B	NM_002093.2	Endogenous
HER2	NM_001005862.1	Endogenous
KLRG1	NM_001329099.1	Endogenous
MDM2	NM_001145337.1	Endogenous
MDM4	NM_001204172.1	Endogenous
MLH1	NM_000249.2	Endogenous
MMP2	NM_004530.5	Endogenous
MMP9	NM_004994.2	Endogenous
MSH2	NM_000251.1	Endogenous
MSH6	NM_000179.1	Endogenous
PMS2	NM_000535.5	Endogenous
RARa	NM_001033603.1	Endogenous
RARb	NM_000965.3	Endogenous
RARg	NM_000966.3	Endogenous
RXRa	NM_002957.4	Endogenous
RXRb	NM_021976.3	Endogenous
RXRg	NM_006917.3	Endogenous
SNAI1	NM_005985.2	Endogenous
SNAI2	NM_003068.3	Endogenous
SRC	NM_005417.3	Endogenous
TIPE1oTNFAIP8L1	NM_001167942.1	Endogenous
TIPE3oTNFAIP8L3	NM_207381.2	Endogenous
TP53	NM_000546.2	Endogenous
TWIST1	NM_000474.3	Endogenous
VCL	NM_014000.2	Endogenous
Wnt1	NM_005430.2	Endogenous
Wnt2	NM_003391.2	Endogenous
Wnt3	NM_030753.3	Endogenous
Wnt5a	NM_003392.3	Endogenous
Wnt7a	NM_004625.3	Endogenous
ZEB1	NM_001128128.1	Endogenous
B2M	NM_004048.2	Housekeeping
GAPDH	NM_001256799.1	Housekeeping
HPRT1	NM_000194.1	Housekeeping
RPL19	NM_000981.3	Housekeeping

Supplementary Table S2. Considering all subjects, higher PGII serum level is associated with H. pylori infection, independently of rs9471643 polymorphism genotype.

rs9471643	*H. pylori-positive*		*H. pylori-negative*		
	**Cases, n**	**PGII, ng/mL §**	**Cases, n**	**PGII, ng/mL §**	***p* ***
C/C	8	20.4 (16.0–37.0)	10	9.8 (7.1–14.9)	0.02
C/G	64	16.1 (13.8–20.5)	26	7.1 (5.1–9.5)	<0.0001
G/G	104	16.0 (10.0–28.7)	58	8.0 (5.7–12.5)	<0.0001
***p* ***		0.36		0.18	

§ Median (IQR); Kruskal-Wallis test. *, P value.

Supplementary Table S3. PGII serum level shows an increasing trend in preneoplastic ACAG diseases and GCs [a].

rs9471643	**Controls**		**FDR-GC**		**ACAG**		**GC**		***p &***
	**n**	**PGII §**	**n**	**PGI I §**	**n**	**PGII §**	**n**	**PGII §**	
C/C	3	6.6 (6.3–6.8)	4	9.8 (8.2–10.9)	3	18.4 (16.6–25.5)	8	22.3 (17.8–40.0)	0.0001
C/G	18	5.8 (4.6–6.8)	31	7.6 (6.7–14.6)	18	9.5 (8.0–12.7)	23	17.4 (10.3–23.2)	<0.0001
G/G	31	5.7 (4.7–7.8)	39	10.0 (7.2–12.5)	41	10.2 (7.1–14.8)	49	17.0 (11.0–34.3)	<0.0001
p *		0.58		0.79		0.08		0.26	

P * and §, p value and Median (IQR),by Kruskal-Wallis test; p *&*, p value by Jonckheere-Terpstra trend test. [a] Patients with dysplasia were not reported because we have only 2 cases.
